# Unexpected gastrointestinal bleeding from the ileocolic artery penetrating the duodenum after hilar cholangiocarcinoma surgery

**DOI:** 10.1002/ccr3.5764

**Published:** 2022-04-18

**Authors:** Sunao Uemura, Hiromichi Maeda, Kazuhiro Hanazaki

**Affiliations:** ^1^ Department of Surgery Kochi Medical School Hospital Nankoku Japan

**Keywords:** gastrointestinal bleeding, intestinal fistula, traffic accident

## Abstract

Cases of bleeding from the ileocolic artery penetrating the duodenum are uncommon, as bleeding from the colonic diverticulum usually spontaneously stops. Herein, this case depicts sudden gastrointestinal bleeding in a patient whose only relevant history was hepaticojejunostomy for hilar cholangiocarcinoma and a previous abdominal surgery for a traffic accident.

## Graphical abstract

Cases of bleeding from the ileocolic artery penetrating the duodenum are uncommon. This case depicts sudden gastrointestinal bleeding in a patient whose only relevant history was hepaticojejunostomy for hilar cholangiocarcinoma and a previous abdominal surgery for a traffic accident.

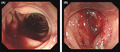

## 1 CASE IMAGE

**FIGURE 1:**
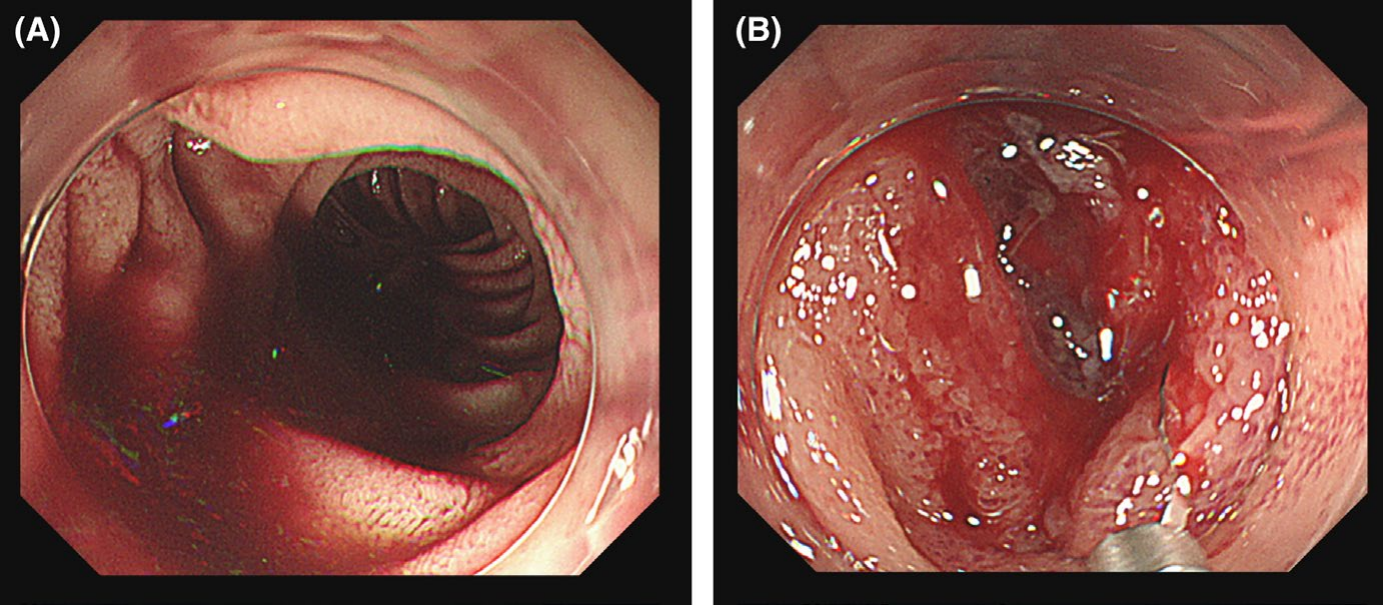
Esophagogastroduodenoscopy shows massive bleeding in the gastrointestinal tract (A) and projectile bleeding on the posterior wall of the descending duodenum (B)

**FIGURE 2:**
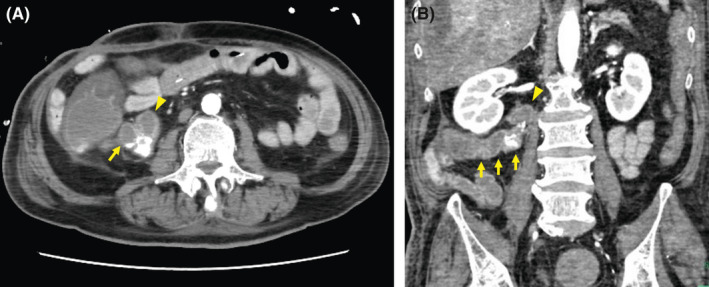
(A) Abdominal contrast‐enhanced computed tomography shows extravasation of contrast medium in the cecum (arrow) and duodenum (arrowhead). (B) The coronal view shows the cecum deforming horizontally (arrows) and in close contact with the duodenum (arrowhead)

**FIGURE 3:**
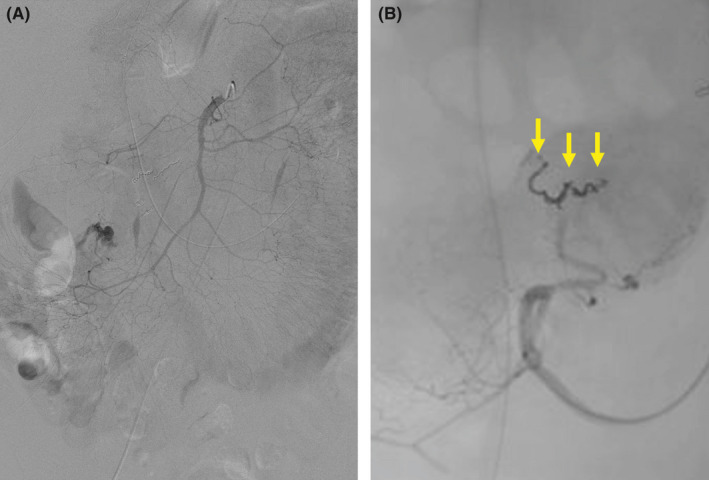
(A) Abdominal angiography reveals extravasation from the ileocolic artery. (B) Selective transcatheter arterial embolization is successfully performed using three coils (Target XL, 2 mm ×3 cm; Stryker, Fremont, CA, USA) (arrows)

A 66‐year‐old man manifested acute massive hematemesis and melena postoperatively. Two months prior, he underwent left hepatectomy with biliary reconstruction for hilar cholangiocarcinoma and recovered from postoperative bile leakage and venous thrombosis. Forty‐six years prior, he underwent abdominal surgery following a traffic accident. Esophagogastroduodenoscopy revealed projectile bleeding on the descending duodenum's posterior wall (Figure 1A,B). However, the source could not be identified on contrast‐enhanced computed tomography (CT) or abdominal angiography. Hematemesis and melena persisted the day after the onset, and subsequent CT revealed extravasation of contrast medium in the cecum and duodenum ((Figure 2A,2B). Repeat angiography revealed extravasation from the ileocolic artery (ICA) ((Figure 3A), and transcatheter arterial embolization (TAE) was successfully performed ((Figure 3B).

Acute lower gastrointestinal bleeding is commonly caused by diverticuli, enterocolitis, and malignant diseases, wherein hematemesis is uncommon.^1^ Particularly, bleeding from the colonic diverticulum usually stops spontaneously,^1^ with a few cases requiring TAE.^2^ Cases of bleeding from the ICA penetrating the duodenum are extremely rare. Herein, the bleeding point was far from the surgical field, and our patient had no underlying diseases. Therefore, the bleeding might have occurred due to a colo‐duodenal fistula from the previous abdominal surgery because the cecum and duodenum were in close contact with each other on preoperative CT.

## CONFLICT OF INTEREST

The authors declare no conflict of interests for this article.

## AUTHOR CONTRIBUTION

Sunao Uemura treated the patient, conceptualized the study, and wrote the original draft. Hiromichi Maeda critically revised the manuscript. Kazuhiro Hanazaki wrote, reviewed, and edited the manuscript.

## ETHICAL APPROVAL

All procedures performed were in accordance with the ethical standards. All authors participated in revising the manuscript and approved the final manuscript.

## CONSENT

Written informed consent was obtained from the patient to publish this case report and the accompanying images.

## Data Availability

The data that support the findings of this study are available from the corresponding author upon reasonable request.
